# Patient reported outcomes measures (PROMs) trajectories after elective hip arthroplasty: a latent class and growth mixture analysis

**DOI:** 10.1186/s41687-022-00503-5

**Published:** 2022-09-09

**Authors:** Davide Golinelli, Alberto Grassi, Dario Tedesco, Francesco Sanmarchi, Simona Rosa, Paola Rucci, Marilina Amabile, Monica Cosentino, Barbara Bordini, Maria Pia Fantini, Stefano Zaffagnini

**Affiliations:** 1 Department of Biomedical and Neuromotor Sciences (DIBINEM), Alma Mater Studiorum - University of Bologna, Via San Giacomo 12, 40126 Bologna, Italy; 2 IIa Clinica Ortopedica e Traumatologica, IRCCS Istituto Ortopedico Rizzoli, Via Pupilli 1, 40136 Bologna, Italy; 3 Directorate-General Personal Care, Health and Welfare, Emilia-Romagna Region, Viale Aldo Moro, 21, 40127 Bologna, Italy; 4 Medical Technology Lab, IRCCS Istituto Ortopedico Rizzoli, Via Pupilli 1, 40136 Bologna, Italy

**Keywords:** Patient-reported outcome measures, PROMs, Quality improvement, Arthroplasty, Hip, Implant registry

## Abstract

**Background:**

Patient-reported outcome measures (PROMs) are an extensively used tool to assess and improve the quality of healthcare services. PROMs can be related to individual demographic and clinical characteristics in patients undergoing hip arthroplasty (HA). The aim of this study is to identify distinct subgroups of patients with unique trajectories of PROMS scores and to determine patients’ features associated with these subgroups.

**Methods:**

We conducted a prospective, cohort study in which PROMs questionnaires (Euro Quality 5 Dimensions 3L, EQ-5D-3L, Euro-Quality-Visual-Analytic-Score, EQ-VAS, Hip disability and Osteoarthritis Outcome Score, HOOS-PS) were administered to patients undergoing elective HA pre-operatively, and at 6 and 12 months after surgery. For each measure, latent class growth analysis and growth mixture models were used to identify subgroups of patients with distinct trajectories of scores. Demografic and clinical predictors of the latent classes in growth mixture model were identified using a 3-step approach.

**Results:**

We found three distinct trajectories for each PROM score. These trajectories indicated a response heterogeneity to the HA among the patients (n = 991). Patient’s gender, ASA score, and obesity were significantly associated with different PROMs trajectories.

**Conclusions:**

We identified three distinct trajectories for each of the three PROMs indicators. Several demographic and clinical characteristics are associated with the different trajectories of PROMs at 6 and 12 months after HA and could be used to identify groups of patients with different outcomes following HA surgery. These findings underline the importance of patient-centered care, supporting the usefulness of integrating PROMs data alongside routinely collected healthcare records for guiding clinical care and maximizing patients’ positive outcomes.

*Trial registration*: Protocol version (1.0) and trial registration data are available on the platform www.clinicaltrial.gov with the identifier NCT03790267, posted on December 31, 2018.

**Supplementary Information:**

The online version contains supplementary material available at 10.1186/s41687-022-00503-5.

## Introduction

Hip arthroplasty (HA) is a widespread and reliable surgical procedure for the end stages of hip pathology, with satisfactory clinical outcomes at 15- to 20-year follow-up [1, 2]. As of today, a dramatic reduction in quality of life (QoL) is a valid indication for total hip replacement, and patients seek so-called high-performance hips to deliver their expectations and aspirations [1]. Before the massive reduction in elective primary and revision HA surgery due to the COVID-19 pandemic [3], the number of HA was rising worldwide [4], because of the higher longevity of the population and the higher incidence of osteoarthritis [5–8]. The increasing volumes of HA procedures entail a greater commitment for healthcare providers, in terms of economic and professional resources necessary to cope with it [8–10].

Although healthcare systems usually monitor healthcare performance and quality indicators, less information is available on how patients experience the process of care and on to what extent it adds value to their lives. Knowledge derived from patient-reported data can be leveraged to develop decision aids and update clinical practice guidelines. Data generated by patients can also contribute to assessing the quality of healthcare pathways especially when combined with other demographic and clinical data derived from routinely collected administrative databases and are increasingly integrated into clinical practice [9].

With a mandate from the Organization for Economic Cooperation and Development (OECD) Health Ministers [10], the OECD launched in 2017 the Patient-Reported Indicators Surveys (PaRIS) Initiative, with the goal of building patient-centered health systems. The PaRIS survey of patients with chronic conditions is aimed to fill this critical information gap, focusing on how patients report about their own health, quality of life, or functional status associated with the healthcare or treatment they have received [10, 11]. The PaRIS Initiative particularly focuses on the systematic collection of PROMs indicators (Euro Quality 5 Dimensions 3L, EQ-5D-3L, Euro Quality Visual Analytic Score, EQ-VAS, and Hip disability and Osteoarthritis Outcome Score, HOOS-PS) in patients undergoing elective hip and knee arthroplasty [10–14, 25].

The IRCCS Rizzoli Orthopedic Institute (IOR), a third-level single-specialty orthopedic hospital based in Bologna, Italy, which is also a member of the International Society of Orthopaedic Centers (ISOC), was selected as one of the pilot centers to launch the PaRIS Initiative in Italy, with the aim to accelerate the adoption and reporting of validated, standardized, internationally comparable patient-reported indicators. About 60% of patients admitted to IOR for joint replacement surgery come from other Italian regions or other countries.

Patients undergoing HA usually experience a significant improvement in PROMs (pain management, usual activities, hip functionality, and self-care) within the first year after surgery and tend to plateau in the following years [15, 16]. Although scientific evidence supports this trend [17], many authors [18–21] have identified subgroups of patients with different recovery trajectories and a diverse range of outcomes, some reporting delayed functional gains, or even a short- and medium-term worsening [22]. Several HA patient characteristics may play a role in determining different PROMs trajectories [23, 24]. It is therefore critical to assess specific subgroups of patients, especially for elective surgery, where a profiled, risk-based management and personalized care can make the difference to reach better outcomes.

The aim of this study is to identify distinct groups of patients with unique score-trajectories for three PROMs indicators (EQ-5D-3L, EQ-VAS, and HOOS-PS), measuring health-related quality of life, pain, symptoms, activity of daily living, and hip functionality, in patients undergoing elective HA, and to determine patients’ features associated with these groups.

## Material and methods

### Study design and data collection

The PaRIS-IOR is a prospective, single-site, cohort study that started on January 1, 2019, and consists of the administration of PROMs questionnaires investigating the quality of life (EQ-5D-3L, and EQ-VAS [18, 19]) and joint-specific functional outcomes (HOOS-PS [24–26]), to patients on the list for elective HA.

PROMs baseline questionnaires were administered to patients awaiting surgery by specifically trained researchers within 30 days before surgery. The follow-up questionnaires were mailed 6 and 12 months after surgery.

IOR hosts the Registry of Orthopedic Prosthetic Implants (RIPO). PROMs data were linked with those routinely collected by the RIPO [27] and other regional administrative data (i.e., hospital discharge records), in order to track patients’ medical history and to define patients’ health profiles.

Patients undergoing elective HA between January 1st and December 31st, 2019, constituted the baseline population. Data included patients’ demographics, pathology leading to joint replacement, type of surgical procedures, in-hospital complications, and the characteristics of the implant. Specifically, we collected and analyzed: (i) the patients’ characteristics and profile, including age and sex distribution, Body Mass Index (BMI), Elixhauser Comorbidity Index (ECI) [28], American Society of Anesthesiologists (ASA) score, Modified-Chronic Disease Score for clinical severity (M-CDS) [29]; (ii) the PROMs questionnaire total scores: EQ-5D-3L score (general range from less than 0, where 0 is a health state equivalent to death and negative values are valued as worse than death to 1, perfect health), EQ-VAS (range 0–100, where 0 is worst and 100 is best), HOOS-PS score (range 0–100, where 0 is worst and 100 is best) for HA patients. The ECI is a comorbidity index based on the International Classification of Diseases (ICD) diagnosis codes. It is obtained as an unweighted count of comorbid conditions [28]. The ASA score is a system for assessing the fitness of patients before surgery. In 1963 the American Society of Anesthesiologists adopted this five-category physical status classification system; a sixth category was later added. The ASA categories (1 to 6) are: Healthy person; Mild systemic disease; Severe systemic disease; Severe systemic disease that is a constant threat to life; A moribund person who is not expected to survive without the operation; A declared brain-dead person whose organs are being removed for donor purposes. The M-CDS [29] is a weighted chronic disease score based on 18 comorbid conditions derived from drug prescriptions that was developed as a prognostic score of 1-year mortality. This score is categorized into 6 classes (0–1, 2, 3–4, 5–6, 7–9, ≥ 10).

Inclusion criteria were age 18–95 years and elective primary hip arthroplasty; exclusion criteria were: severe cognitive impairment; arthroplasty for musculoskeletal cancer; patient not eligible for surgical procedures; hip arthroplasty in the 12 months prior to enrollment. The detailed study protocol, inclusion and exclusion criteria and other information are described in a previous publication [30]. This study follows the STROBE reporting guidelines for observational studies [31, 32].

### Instruments

The choice of using the selected PROMs measures was based on consensus from the OECD’s PaRIS Initiative group, as reported in a previous publication [25, 30]. The validated EQ-5D-3L Italian version was used in this study [33, 34]. The EQ-5D-3L health status and quality-of-life measure is composed of five items (mobility, self-care, usual activity, pain/discomfort, and anxiety/depression) [34]. The 3L version describes health on three levels (no problems, some problems, and a lot of problems) resulting in 243(3^5^) health states [33, 34]. The EQ-5D-3L index is calculated from the scores of the five dimensions, ranging from − 0.594 (worst) to 1.0 (best). Moreover, the EQ-5D-3L includes a VAS for rating of overall health status from 0 (worst imaginable health) to 100 (best imaginable health) [35].

The Italian validated version of the Physical function Short form of the Hip Disability and Osteoarthritis Outcome Survey (HOOS-PS) was used in this study [26]. It consists of 5 items (sitting, descending stairs, getting in/out of the bath/shower, running, twisting, or pivoting on the loaded leg) rated on a five-point Likert scale (none, mild, moderate, severe, extreme), where 0 indicates no problems and 4 extreme problems. The reliability of the 5 items was 0.80 (Cronbach’s alpha [36]. The total score can be transformed to a 0–100 scale, with 0 indicating the worst problems and 100 indicating no problems. The HOOS-PS has been used in subjects with hip disability with or without hip osteoarthritis and in patients with hip osteoarthritis pre- and postoperative total hip replacement. It is suitable for use in research as a disease-specific questionnaire [26, 37].

### Statistical analysis

Baseline demographic and clinical characteristics were summarized using mean and standard deviation, median and interquartile range, or absolute and percentage frequencies, as appropriate. To determine whether patients completing the study questionnaires at 6 and 12 months were representative of the baseline sample, patients lost to follow-up and completers were compared at 12 months on age, gender, BMI, ASA score, region of residence, and primary diagnosis. Information about variable distributions and missing data can be found in the Additional file 1. Continuous variables were compared between groups using t-test and categorical variables using chi-square test or Fisher’s exact test, as appropriate. The significance level was set to 0.05.

#### Latent class growth analysis and growth mixture model

Latent class growth analysis (LCGA) was carried out as an initial modelling step to identify subgroups of patients with different trajectories of functioning and quality of life from pre-surgery and to 12 months following total hip replacement.

LCGA is a special type of Growth Mixture Modeling, whereby the variance and covariance estimates for the growth factors within each class are assumed to be fixed to zero [38]. By this assumption, all individual growth trajectories within a class are homogeneous. This technique allows the user to classify distinct subgroups that follow a similar pattern of change over time, hence it is appropriate for analyzing longitudinal data [39, 40]. Other longitudinal methodologies, such as conventional growth models, assume that individuals come from a single population and that a single trajectory can adequately summarize the entire population. Moreover, they assume that covariates that affect the growth factors influence individuals in the same way. However, we have theoretical reasons to assume that in a clinical population (and specifically among elderly patients) a single growth trajectory would be an oversimplification of the complex growth pattern that characterized changes among members of different groups.

LCGA can accommodate missing data at 6 and/or 12 months using the full information maximum likelihood algorithm (Additional file 1: Figure S1), thus allowing the user to define trajectories for the full set of patients [40, 41].

LCGA requires that assumptions are met concerning within-class conditional normality, a properly specified mean and covariance structure, the linearity of effects of exogenous predictors, a missing at random (MAR) mechanism underlying missing data, and that sample individuals are independent and self-weighted [38–41].

Standard inferential model fit indices were used to identify the best fitting models. Model fit indices included the Akaike Information Criteria (AIC) and Bayesian Information Criterion (BIC). These indices have no predefined cut-offs and can only be interpreted when comparing different models. Lower AIC and BIC indicate a better model fit. Other indices included entropy (values close to 1.0 denote excellent fit), no less than 1% total count in a class, and high posterior probabilities. In addition, Vuong-Lo-Mendell-Rubin likelihood ratio test was used to determine the number of classes. The final model was based on statistical considerations and clinical meaningfulness.

Growth mixture models were then used to estimate separate growth models for each latent class identified by the LCGA. Individuals were assigned to the most likely latent class based on posterior probabilities [32]. Lastly, we analysed the demographic and clinical predictors of the latent classes in GMM using a 3-step approach [42]. This approach takes into account the measurement error in the class assignment process and prevents defining class membership from being influenced by covariates. Specifically, we included the following demographic and clinical variables that are routinely recorded in the administrative databases or in the registry: age, sex (male as reference category), BMI (normal weight/underweight as the reference category), diagnosis (primary coxarthrosis vs. other diagnoses as reference categories), ASA score (1 as the reference category, 2, ≥ 3). Multicollinearity of variables was assessed using the variance inflation factor (VIF). No adjustment was made in the analyses for multiple outcomes.

Patients were cross-classified according to the trajectory group for two PROMs indicators: HOOS-PS and EQ-5D-3L. The former was chosen to account for patients’ reported functionality and mobility, while the latter for patients’ reported quality of life. This choice was made to investigate the distribution of patient reported QoL and functionality scores within the study population. We then analyzed differences in demographic and clinical characteristics between the sub-group of patients showing worse reported outcomes in both PROMs indicators.

All statistical analyses were performed using SPSS, version 25.0, R, version 4.1.0 and the package lcmm [41], and MPlus version 8.7.

### Protocol registration

Protocol version (1.0) and trial registration data are available on the platform www.clinicaltrial.gov with the identifier NCT03790267, posted on December 31, 2018.

## Results

### Study population

**Fig. 1:**
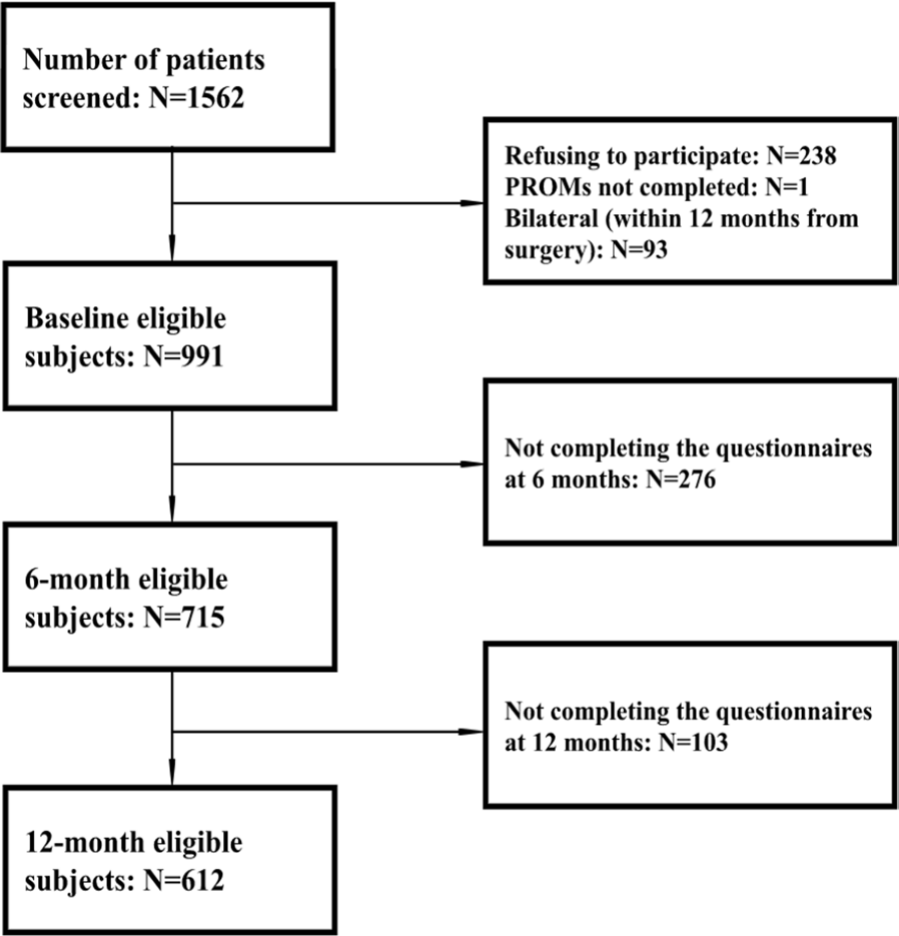
Flow chart of the study population

During the study period, 1562 patients underwent HA at the IOR. After excluding non-eligible patients (*n* = 239), patients refusing to participate in the study (*n* = 238), missing baseline PROMs (*n* = 1), and patients with bilateral surgery where the most recent surgery was already included (*n* = 93) the study population (Table 1, Fig. 1) consisted of 991 HA patients.

**Table 1:** Baseline characteristics of the study population (n = 991) and preoperative and postoperative PROMs

	n	%	Mean	SD
Mean age, years	991		60.4	13.9
Sex				
Female	483	48.7		
Male	508	51.3		
BMI, n (%)				
Normal weight/underweight	340	35.6		
Overweight (BMI ≥ 25, < 30)	402	42.1		
Obese (BMI ≥ 30)	213	22.3		
Diagnosis				
Primary coxarthrosis	696	70.2		
Other	295	29.8		
ASA score				
1	235	25.5		
2	526	57.1		
3	160	17.4		
Incision				
Anterior	294	29.9		
Lateral	623	63.4		
Posterior-lateral	66	6.7		
Head diameter				
≤ 32 mm	435	43.9		
> 32 mm	556	56.1		
Length of stay, d	991		6.8	2.6
Residents in Emilia-Romagna Region	459	46.3		
M-CDS	459			
0–1	124	27.0		
2–4	189	41.2		
5–6	68	14.8		
7–9	37	8.1		
≥ 10	41	8.9		
PROMs baseline score				
EQ-5D-3L	991		0.5	0.2
EQ-VAS	991		52.2	18.4
HOOS-PS	990		54.0	17.0
PROMs 6-months score				
EQ-5D-3L	715		0.8	0.2
EQ-VAS	715		77.9	15.4
HOOS-PS	715		83.3	15.7
PROMs 12-month score				
EQ-5D-3L	612		0.8	0.2
EQ-VAS	612		79.3	16.9
HOOS-PS	612		84.9	15.4

Available only for patients residing in Emilia Romagna region (N = 459)

M-CDS, Modified-Chronic Disease Score

Table 1 shows the baseline patient characteristics. Mean age was 60.4 years (SD = 13.9) and 48.7% were female. Overall, the mean preoperative PROMs were: 0.5 (SD = 0.2) for EQ-5D-3L score, 52.2 (SD = 18.4) for EQ-VAS, and 54.0 (SD = 17.0) for HOOS-PS. Of those included, complete PROMs data were available at 6 months for 715 patients (72.1%), and at 12 months for 612 patients (61.7%). The mean postoperative PROMs at 12 months were 0.8 (SD = 0.2) for EQ-5D-3L score, 79.3 (SD = 16.9) for EQ-VAS, and 84.9 (SD = 15.4) for HOOS-PS.

The mean postoperative PROMs at 6 and 12 months were 0.8 (SD = 0.2) and 0.8 (SD = 0.2) for EQ-5D-3L score, 77.9 (SD = 15.4) and 79.3 (SD = 16.9) for EQ-VAS, and 83.3 (SD = 15.7) and 84.9 (SD = 15.4) for HOOS-PS.

Patients assessed at 12 months had similar baseline characteristics compared with those who did not complete the 12-month survey (Additional file 1: Table S1), except for an older age (mean age of 62.3 [SD = 13.0] vs. 57.4 [SD = 14.7], of non-completers, *p* < 0.001), region of residence (51.6% vs. 37.7% resident in Emilia-Romagna, *p* < 0.001) and primary diagnosis (73.5% vs. 64.9% of primary coxarthrosis, *p* = 0.004). Moreover, completers had significantly higher mean baseline scores than non-completers on PROM measures: mean EQ-5-3LD 0.53 [SD = 0.22] vs. 0.48 [SD = 0.23], *p* = 0.001; mean EQ-VAS 53.7 [SD = 17.9] vs. 49.7 [SD = 19.0], *p* = 0.001; mean HOOS-PS 53.7 [SD = 17.9] vs. 51.8 [SD = 17.7], p = 0.002.

### Model selection and characterization of trajectories

We used LCGA to select the number of trajectories for each PROM measure (Additional file 1: Table S2–S7).

The baseline frequency distribution was normal for EQ-VAS and HOOS-PS scores and bi-modal for EQ-5D-3L, thereby violating the underlying assumption of normality for this scale. Therefore, for EQ-5D-3L we estimated a latent-class mixed-effect model for continuous non-Gaussian outcomes (R function lcmm). Results were overlapping with those based on the assumption of normality (R function hlme) [41]. Thus, in the remainder of the manuscript we report results based on the assumption of normality for all the PROMs measures. We tested three different covariance structures (autoregressive, Brownian motion and unstructured) and found that results were very similar. The following results assume that covariance is unstructured.

The LCGA with three classes was chosen over the 2-class groups (Additional file 1: Tables S2–S7) to achieve a balance between model parsimony and a better description of PROMs patterns for each PROMs indicator. The 3-class model produced three distinct PROMs trajectories including at least 3% of cases in the smallest class. The posterior probability of membership for each allocated class ranged between 0.72 and 0.85 for EQ-VAS, between 0.91 to 0.95 for EQ-5D-3L and 0.79 to 0.86 for HOOS-PS (Additional file 1: Tables S5–S7). All indicators of the HOOS-PS converged in suggesting the 3-class solution, while for the EQ-VAS and EQ-5D-3L we selected the model providing a best description of PROMs patterns based on clinical interpretation. The 4-class models were discarded because they did not result in a better fit to the data.

**Fig. 2:**
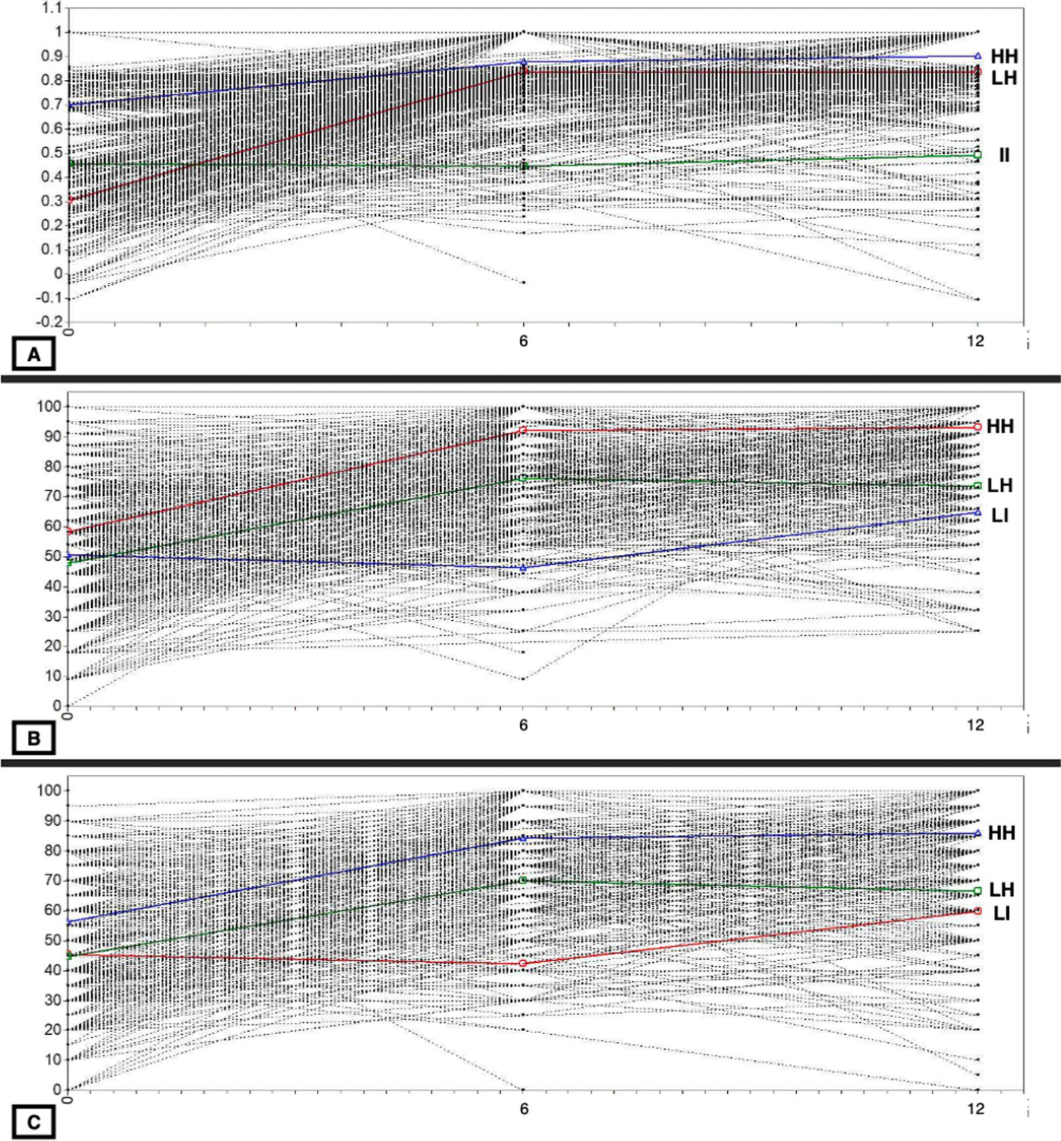
Spaghetti plots of individual trajectoriess for EQ-5D-3L (**A**), HOOS-PS (**B**), and EQ-VAS (**C**) scores, and estimated trajectories using Latent Class Growth Analysis. HH, High-High PROMs trajectory; II, Intermediate-Intermediate PROMs trajectory; LH, Low–High PROMs trajectory; LI, Low-Intermediate PROMs trajectory

We then estimated the GMM model, in which the intercept and the slopes are allowed to vary within classes. A spaghetti plot with the individual trajectories for the three PROMs measures and the trajectories estimated using GMM is provided in Fig. 2.

EQ-5D-3L trajectories. The first trajectory included 503 (50.8%) patients starting with higher pre-surgery EQ-5D-3L score, improving at 6 months, and maintaining a stable score at 12 months (high-high class, HH). The second trajectory included 448 (45.2%) patients starting with low EQ-5D-3L scores, strongly improving at 6 months, and remaining stable at 12 months (low–high class, LH). The third trajectory included a small group of 40 (4%) individuals, starting with an intermediate score, that remained stable at 6 months and 12 months (intermediate II).

HOOS-PS trajectories. The first trajectory included 618 (62.4%) patients starting with higher pre-surgery HOOS-PS, improving at 6 months, and maintaining a stable score at 12 months (HH class). The second trajectory included 322 (32.6%) patients starting with low HOOS-PS scores, strongly improving at 6 months, and stabilizing afterwards (LH class). The third trajectory included a small group of 50 (5%) individuals, starting with a low HOOS-PS score, that remained stable at 6 months and then slightly improved at 12 months (class LI).

EQ-VAS trajectories. The first trajectory included 715 (72.2%) patients starting with higher pre-surgery EQ-VAS, improving at 6 months, and maintaining a stable score at 12 months (HH class). The second trajectory included 40 (4%) patients starting with low EQ-VAS scores, remaining stable at 6 months, and slightly improving at 12 months (LI class). The third trajectory included a small group of 235 (23.8%) individuals, starting with an intermediate EQ-VAS score, that remained stable at 6 months and then slightly declined at 12 months (LH class).

Patients were cross-classified according to the trajectory group for HOOS-PS and EQ-VAS to determine whether the results were consistent across PROMs indicators (Additional file 1: Tables S8 and S9). 373 patients fell in the HH trajectory across the two PROMs indicators, 191 fell in the LH trajectory and 15 patients fell in the worst trajectory group for both indicators (II/LI). The comparison between the subgroup of patients showing worse reported outcomes in both HOOS-PS and EQ-5D-3L trajectories with the rest of sample (Table S9) showed that the 15 patients were significantly older, and with higher ASA scores.

### Socio-demographic and clinical characteristics of patients in the trajectory groups

EQ-5D-3L trajectories (Table 2). Patients assigned to the LH trajectory based on posterior probabilities estimated by GMM were more likely to be female (OR = 2.06, 95% CI 1.47–2.89, *p* < 0.001), younger (OR = 0.98, 95% CI 0.97–0.99, *p* = 0.041), to present obesity (OR = 2.39, 95% CI 1.52–3.72, *p* < 0.001) and to have an ASA score of 2 (OR = 1.83, 95% CI 1.22–2.76, *p* = 0.004) or ≥ 3 (OR = 3.60, 95% CI 1.94–6.68, *p* < 0.001) than those in HH trajectory. Vice versa, patients in the II trajectory group were more likely to have an ASA score ≥ 3 (OR = 9.30, 95%CI 1.86–46.4, *p* = 0.007) than those in HH trajectory.

**Table 2:** Results of the multinomial logistic regression for EQ-5D-3L

Covariates	LH versus HH			II versus HH		
	Odds ratio	95% CI	*p* value	Odds ratio	95% CI	*p* value
Sex						
Females	2.061	1.468–2.893	**< 0.001**	1.985	0.837–4.708	0.120
Males	–	–	**–**	–	–	–
Age	0.984	0.970–0.999	**0.041**	0.992	0.951–1.034	0.698
BMI						
Obese	2.375	1.519–3.715	**< 0.001**	2.746	0.945–7.982	0.063
Overweight	1.314	0.909–1.901	0.146	1.842	0.720–4.714	0.203
Normal weight/underweight	–	–	–	–	–	–
ASA score						
3	3.602	1.943–6.679	**< 0.001**	9.300	1.864–46.397	**0.007**
2	1.834	1.218–2.761	**0.004**	1.462	0.385–5.551	0.577
1	–	–	**–**	–	–	–
Diagnosis						
Primary coxarthrosis	1.281	0.885–1.854	0.189	1.065	0.438–2.594	0.889
Other diagnosis	–	–	–	–	–	–

Bold indicates statistically significant results (i.e. those where* p* < 0.05)

HH, High-High PROMs trajectory; II, Intermediate-Intermediate PROMs trajectory; LH, Low–High PROMs trajectory

HOOS-PS trajectories (Table 3). Patients in the LH trajectory group were more likely to be female (OR = 3.04, 95% CI 1.73–5.35, *p* < 0.001), to present obesity (OR = 4.45, 95% CI 2.05–9.63, *p* < 0.001) or overweight (OR = 2.50, 95% CI 1.30–4.84, *p* = 0.006), have an ASA score ≥ 3 (OR = 4.60, 95% CI 1.51–14.00, *p* = 0.007) and with a diagnosis of primary coxarthrosis (OR = 2.32, 95% CI 1.25–4.29, *p* = 0.008) than those in the HH trajectory group. Vice versa, patients with the LI trajectory group were more likely to be female (OR = 3.79, 95% CI 1.67–8.56, *p* = 0.001) and to have an ASA score ≥ 3 (OR = 6.80, 95%CI 1.14–40.58, *p* = 0.036) than those in the HH trajectory group.

**Table 3:** Results of the multinomial logistic regression for HOOS-PS

Covariates	LI versus HH			LH versus HH		
	Odds ratio	95% CI	*p* value	Odds ratio	95% CI	*p* value
Sex						
Females	3.785	1.674–8.555	**0.001**	3.043	1.730–5.353	**< 0.001**
Males	–	–	–	–	–	**–**
Age	1.039	0.995–1.086	0.082	1.013	0.984–1.043	0.391
BMI						
Obese	2.799	0.958–8.181	0.060	4.445	2.051–9.631	**< 0.001**
Overweight	1.550	0.624–3.847	0.345	2.504	1.296–4.839	**0.006**
Normal weight/underweight	–	–	–	–	–	**–**
ASA score						
3	6.795	1.138–40.580	**0.036**	4.596	1.509–14.003	**0.007**
2	2.961	0.591–14.819	0.187	1.792	0.775–4.143	0.173
1	–	–	–	–	–	**–**
Diagnosis						
Primary coxarthrosis	1.596	0.632–4.031	0.322	2.316	1.250–4.292	**0.008**
Other diagnosis	–	–	–	–	–	**–**

Bold indicates statistically significant results (i.e. those where* p* < 0.05)

HH, High-High PROMs trajectory; LH, Low–High PROMs trajectory; LI, Low-Intermediate PROMs trajectory

EQ-VAS trajectories (Table 4). Patients in the LH trajectory group based on posterior probabilities estimated by GMM were more likely to be female (OR = 3.88, 95% CI 1.85–8.14, *p* < 0.001) and to have an ASA score of 2 (OR = 3.15, 95% CI 1.10–9.00, *p* = 0.033) or ≥ 3 (OR = 12.62, 95% CI 2.95–53.94, *p* = 0.001) than those in the HH trajectory group.

**Table 4:** Results of the multinomial logistic regression for EQ-VAS

Covariates	LI versus HH			LH versus HH		
	Odds ratio	95% CI	*p* value	Odds ratio	95% CI	*p* value
Sex						
Females	1.271	0.509–3.176	0.607	3.883	1.852–8.144	**< 0.001**
Males	–	–	–	–	–	–
Age	1.004	0.952–1.060	0.877	0.979	0.943–1.016	0.261
BMI						
Obese	1.985	0.617–6.387	0.250	2.641	0.994–7.015	0.051
Overweight	1.369	0.484–3.870	0.554	1.804	0.821–3.962	0.142
Normal weight/underweight	–	–	–	–	–	–
ASA score						
3	5.580	0.896–34.764	0.065	12.622	2.953–53.944	**0.001**
2	0.916	0.231–3.623	0.900	3.146	1.100–8.996	**0.033**
1	–	–	–	–	–	–
Diagnosis						
Primary coxarthrosis	0.850	0.279–2.590	0.775	1.340	0.580–3.100	0.493
Other diagnosis	–	–	–	–	–	–

Bold indicates statistically significant results (i.e. those where* p* < 0.05)

HH: High-High PROMs trajectory; LH: Low–High PROMs trajectory; LI: Low-Intermediate PROMs trajectory

The VIF ranged from 1.05 to 1.59, raising no concerns about multicollinearity.

### Impact of COVID-19 pandemic on study procedures.

We compared the completion rate and the PROMs scores between patients who completed the 6-month and 12- months follow-up before and during the lockdown. Results indicate that at 6 months the completion rate of PROMs questionnaires declined significantly (Tables S10a and S10b). Moreover, the mean HOOS-PS score was significantly higher among those who completed the 6-month follow-up during the Italian lockdown. No difference in the PROMs mean scores and completion rate was found at 12 months.

## Discussion

This study identified three outcome trajectories of quality of life and functioning among patients who underwent HA, and the characteristics associated with them. These trajectories were consistent among the three PROMs measures, with one trajectory (HH) accounting for most patients in each scale and showing an increasing trend in quality of life and functioning for these patients at 6 months after surgery, followed by a plateau at 12 months. The second trajectory (LH) was more heterogenous among the PROMs indicators, but, overall, it was characterized by low baseline scores and significant improvements at 6 and 12 months after surgery. The third trajectory started with lower baseline scores and was characterized by a modest improvement only at 12 months. This trajectory, accounting for a smaller number of patients, showed that a share of patients undergoing HA do not fully benefit from surgery. Our findings suggest that female gender, obesity, and a high ASA score (≥ 3) were associated with the trajectory characterized by worse PROMs at the time of HA surgery, both in terms of quality of life and functioning.

Overall, baseline characteristics and PROMs score of the study population, both pre and post operatively, are in line with previous studies [43–45], although data were collected at slightly different time points. In fact, this study has an important strength in the collection of PROMs at 6 months after surgery, differently from other studies which investigated outcomes at longer-term time-points (12 or 24 months) [11–13, 16, 22, 41–45]. Therefore, it provides insights into the medium-term PROMs and the effect of surgical intervention across different patient groups. For instance, regarding the patient-reported hip functionality (HOOS-PS), we observed a larger group of 'quick responders' who rapidly improved 6 months after surgery and a group of 'late responders', who instead reached better levels of functioning only after 12 months. This offers useful hints for the clinical management of these patients.

More specifically, we found that the highest proportion of patients was admitted to surgery with high PROMs scores, slightly improved at 6 months after surgery and preserved adequate levels of functioning and quality of life at 12 months. This kind of trajectory is frequently reported for patients undergoing hip surgery [44, 46]. However, unlike much of the available literature [11–13, 16, 22, 43, 44], this study underlines the importance of analyzing 6-months PROMs trends to identify the medium-term effects of HA surgery on patients’ QoL and hip-specific functioning. A second subgroup of patients started from low PROMs scores, but strongly improved at 6 months and then preserved high levels of functionality and QoL still at 12 months after surgery. This finding is consistent with the literature [11–13, 16, 22, 43–45, 47], as it is common for HA patients to exhibit improved functioning and quality of life after surgery and then reach a plateau. However, we found that a small number of patients, admitted to HA surgery with low-intermediate PROMs scores, did not show any significant improvement at 6 months, and only a minimal improvement at 12 months after surgery. This subgroup of patients, showing worse reported outcomes in both HOOS-PS and EQ-5D-3L trajectories was older and had higher ASA score than the rest of the study population.

Our analyses showed some baseline demographic and clinical characteristics associated with each trajectory. Obesity was associated with a higher likelihood of being in the trajectory (LH) starting with lower baseline functioning scores (HOOS-PS). This is in line with evidence from the literature [44, 48] suggesting that obesity is a strong predictor of conditions that require HA (e.g., osteoarthritis) and poor outcomes in terms of joint’s functioning.

Our results also indicate that an ASA score ≥ 3 is related to the trajectories starting with worse PROMs scores and is associated with the subgroup of patients undergoing HA who do not fully benefit from surgery. This finding confirms the body of evidence on the importance of the ASA score for stratifying patients at different risk of worse outcomes after surgery [49–51]. The use of the ASA score is indeed widespread among healthcare professionals to assess patients' eligibility for surgery [50, 51]. Our study suggests that this score, combined with other indicators, could be implemented to define a patient's functional prognosis as well as the expected QoL improvement after the intervention, thus allowing patients and surgeons to make the most sensible/appropriate choice, relying on a commonly used tool.

Notably, being female is related both to the worst baseline PROMs and to a higher probability of improvement for each of the considered scores. Female subjects had lower pre-surgery scores and showed the largest improvement 6 months after the intervention; therefore, they benefited the most from the arthroplasty. A possible explanation is that females are better responders to surgery—and more compliant to rehabilitation programs—compared with males [52–56].

### Strengths and limitations of the study

One strength of our study is the latent class and growth modelling strategy, that allowed us to capture information about inter-individual differences in intra-individual change. We argue that this person-centered approach aimed at classifying individuals into distinct classes is more informative from the clinical standpoint than a variable-centered approach that seeks to identify significant predictors of outcomes. In addition, our strategy allowed to estimate the PROMs trajectories taking into account the heterogeneity of individuals within classes and to identify the predictors of class assignment allowing for a measurement error in the class assignment.

The study population was recruited in a large-volume single third-level mono-specialty hospital that is a reference center in Italy for orthopedics and bone diseases. Our findings are therefore based on a highly selected population. Given the analytical approach of this study, our findings can only be generalized to patients with the same inclusion/exclusion criteria, therefore, evidence from studies carried out in different populations and in different healthcare facilities are needed to confirm and generalize our findings.

Moreover, our results could be affected by the patient's attrition. Specifically, patients lost at follow-up differed on some characteristics from those assessed at 12 months (e.g., age), which may constitute a possible bias and limit the study’s internal validity. Still, our attrition rate is similar to the one reported in other similar studies [11, 17, 22, 57]. We also identified several factors related to loss at 6- and 12-month follow-up. However, we cannot exclude that other unobserved variable are informative of the missingness process (e.g., educational level and socio-economic status).

Furthermore, PROMs assessment at only three times points limited our ability to capture early improvements or worsening or complex trajectories of change. The wide CIs for some of the comparisons are due to the small number of patients in some subgroups and this limited our power to detect significant differences.

No adjustment to the probability level for multiple outcomes was undertaken, given the exploratory nature of the study.

Lastly, some patients undergoing HA surgery in 2019 have completed their follow-up questionnaires during the first year of the COVID-19 pandemic. This resulted in a lower completion rate (67.2% vs. 74.6%) and higher HOOS-PS scores among those who had the 6-month follow-up during the lockdown than the rest of the sample. However, our analyses (Additional file 1) showed no significant differences concerning the 12-month follow-up, which partly mitigates the possible impact of the social distancing measures enforced by the Italian government during the period March–May 2020 (i.e., lockdown).

## Conclusions

The PaRIS-IOR study represents the first large systematic collection of PROMs in patients undergoing arthroplasty in Italy and has important implications in targeting the factors affecting patient-reported outcomes after joint arthroplasty.

We identified three distinct trajectories for each of the PROMs indicators that summarize the response heterogeneity to the surgical procedure of HA patients. Females seem to benefit the most from HA, while an ASA score ≥ 3 and obesity are associated with poorer baseline PROMs scores and trajectories at 6 and 12 months after HA. Further studies are needed to confirm whether similar trajectories can be found in other samples of patients undergoing elective HA. These findings also underline the importance of patient-centered care, supporting the usefulness of integrating PROMs data alongside routinely collected healthcare records for guiding clinical care and maximizing patient outcomes.

## Supplementary Information

**Additional file 1**. Supplementary material.

**Additional file 2**. R Script for LCGA.

## Data Availability

The data used in the study are controlled by Rizzoli Orthopedic Institute and cannot be shared publicly. However, aggregated, and anonymized data are available upon specific request to the corresponding authors. Interested researchers can replicate our study findings by contacting the authors or the Rizzoli Orthopedic Institute.

## Code availability

The commented script is available as Additional file 2 (R Script for LCGA).
