# Supplementary material for: Patient reported outcomes measures (PROMs) trajectories after elective hip arthroplasty: a latent class and growth mixture analysis
Source: J Patient Rep Outcomes. 2022 Sep 9;6:95. doi: 10.1186/s41687-022-00503-5 (PMC9462642; doi:10.1186/s41687-022-00503-5)
Supplement: Supplementary file 1 — Additional file 1. Supplementary material. [file 41687_2022_503_MOESM1_ESM.docx]

**Supplemental material**

**Missing data treatment information**

We employed Full Information Maximum Likelihood (FIML), to estimate missing data of outcome variables. Missing values of covariates were not imputed. Missing data patterns are represented in Figure S1.

**Figure S1. Missing value pattern of study variables.**


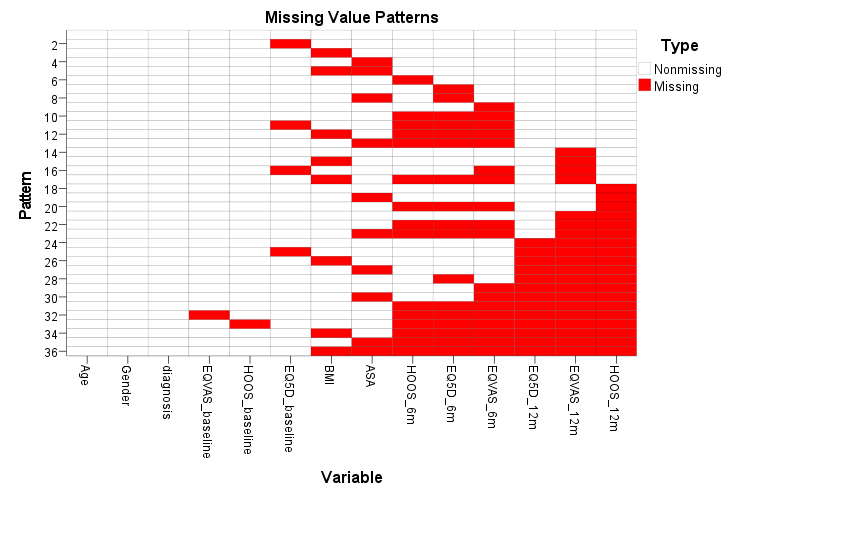


**Table S1: Patients' characteristics. Comparison between completers vs lost to 12-month follow-up.**

**​​**

|  | | **Lost to follow-up** | | | | **Completers** | | | |  |
| --- | --- | --- | --- | --- | --- | --- | --- | --- | --- | --- |
|  | | ***n*** | **%** | **Mean** | **SD** | ***n*** | **%** | **Mean** | **SD** | **p-value** |
| **Mean Age**, *years* | | *379* |  | 57.4 | 14.7 | 612 |  | 62.3 | 13.0 | < 0.001 |
| **Sex**, | |  |  |  |  |  |  |  |  | 0.341 |
|  | Female | *192* | 50.7 |  |  | 291 | 47.5 |  |  |  |
|  | Male | *187* | 49.3 |  |  | 321 | 52.5 |  |  |  |
| **BMI** | |  |  |  |  |  |  |  |  | 0.097 |
|  | normal weight/underweight | *135* | *37.5* |  |  | 205 | 34.5 |  |  |  |
|  | overweight | *136* | *37.8* |  |  | 266 | 44.7 |  |  |  |
|  | obese | *89* | *24.7* |  |  | 124 | 20.8 |  |  |  |
| **Diagnosis** | | | | | |  |  |  |  | 0.004 |
|  | Primary coxarthrosis | *246* | *64.9* |  |  | 450 | 73.5 |  |  |  |
|  | Other | 133 | 35.1 |  |  | 162 | 26.5 |  |  |  |
| **Incision** | |  |  |  |  |  |  |  |  | 0.516 |
|  | Anterior | *119* | 31.6 |  |  | 175 | 28.9 |  |  |  |
|  | Lateral | 236 | 62.6 |  |  | 387 | 63.9 |  |  |  |
|  | Posterior-Lateral | 22 | 5.8 |  |  | 44 | 7.3 |  |  |  |
| **Head diameter** | |  |  |  |  |  |  |  |  | 0.028 |
|  | ≤32 mm | *183* | 48.3 |  |  | 252 | 41.2 |  |  |  |
|  | >32 mm | *196* | *51.7* |  |  | 360 | 58.8 |  |  |  |
| **ASA score** | | | | | |  |  |  |  | 0.165 |
|  | 1 | *99* | *28.7* |  |  | 136 | 23.6 |  |  |  |
|  | 2 | *193* | *55.9* |  |  | 333 | 57.8 |  |  |  |
|  | 3 | *53* | *15.4* |  |  | 107 | 18.6 |  |  |  |
| **M-CDS*** | | *143* |  |  |  | 316 |  |  |  | 0.789 |
|  | 0-1 | *42* | *29.4* |  |  | 82 | 25.9 |  |  |  |
|  | 2-4 | *53* | *37.1* |  |  | 136 | 43.0 |  |  |  |
|  | 5-6 | *23* | *16.1* |  |  | 45 | 14.2 |  |  |  |
|  | 7-9 | *11* | *7.7* |  |  | 26 | 8.2 |  |  |  |
|  | >=10 | *14* | *9.8* |  |  | 27 | 8.5 |  |  |  |
| **In-hospital complications** | | | | | |  |  |  |  | 0.935 |
|  | no | *377* | *99.5* |  |  | 609 | 99.5 |  |  |  |
|  | yes | *2* | *0.5* |  |  | 3 | 0.5 |  |  |  |
| **Lenght of stay, days** | |  |  | 6.5 | 2.1 |  |  | 7.0 | 2.8 | < 0.01 |
| **Residents in RER** | | *143* | 37.7 |  |  | 316 | 51.6 |  |  | < 0.001 |
| **PROMs baseline score** | |  |  |  |  |  |  |  |  |  |
| **EQ-5D-3L** | | *379* |  | 0.48 | 0.23 | *612* |  | 0.53 | 0.22 | 0.001 |
| **EQ-VAS** | | *379* |  | 49.7 | 19.0 | *612* |  | 53.7 | 17.9 | 0.001 |
| **HOOS-PS** | | *379* |  | 51.8 | 17.7 | *612* |  | 55.3 | 16.5 | 0.002 |

***M-CDS=Modified Chronic Disease Score**

**Table S2. Goodness of fit parameters of the LCGA and GMM models for HOOS-PS.**

| Model | Classes | | LL | npar | AIC | BIC | Entropy | A-LRT | p-value |
| --- | --- | --- | --- | --- | --- | --- | --- | --- | --- |
| LGCA | | 1 | -9598.370 | 6 | 19208.741 | 19238.127 |  |  |  |
| LCGA | | 2 | -9329.524 | 10 | 18679.047 | 18728.024 | 0.763 | 518.887 | <0.001 |
| LCGA | | 3 | -9253.883 | 14 | 18535.767 | 18604.334 | 0.714 | 145.990 | 0.021 |
| LCGA | | 4 | -9212.409 | 18 | 18460.818 | 18548.976 | 0.752 | 80.048 | 0.182 |
| GMM | | 3 | -9130.801 | 26 | 18313.603 | 18440.943 | 0.583 | 137.607 | 0.240 |

Labels: LL = Log Likelihood, npar = Number of Free Parameters, AIC = Akaike’s Information Criterion, BIC = Bayesian Information Criterion, L2 = Likelihood Ratio Chi-Square, A-LRT = Lo-Mendell-Rubin Adjusted Likelihood Ratio Test. LCGA = latent class growth analysis, GMM = growth mixture model.

**Table S3. Goodness of fit parameters of the LCGA and GMM models for EQ-VAS.**

| Model | Classes | LL | npar | AIC | BIC | Entropy | A-LRT | p-value |
| --- | --- | --- | --- | --- | --- | --- | --- | --- |
| LCGA | 1 | -9788.728 | 6 | 19589.455 | 19618.842 |  |  |  |
| LCGA | 2 | -9607.597 | 10 | 19235.194 | 19284.171 | 0.733 | 349.590 | <0.001 |
| LCGA | 3 | -9560.055 | 14 | 19148.110 | 19216.678 | 0.695 | 91.759 | 0.165 |
| LCGA | 4 | -9534.220 | 18 | 19104.441 | 19192.600 | 0.595 | 49.862 | 0.511 |
| GMM | 3 | -9467.493 | 26 | 18986.986 | 19114.326 | 0.546 | 70.693 | 0.240 |

Labels: LL = Log Likelihood, npar = Number of Free Parameters, AIC = Akaike’s Information Criterion, BIC = Bayesian Information Criterion, L2 = Likelihood Ratio Chi-Square, A-LRT = Lo-Mendell-Rubin Adjusted Likelihood Ratio Test., LCGA = latent class growth analysis, GMM = growth mixture model.

**Table S4. Goodness of fit parameters of the LCGA and GMM models for EQ-5D -3L.**

| Model | Classes | LL | npar | AIC | BIC | Entropy | A-LRT | p-value |
| --- | --- | --- | --- | --- | --- | --- | --- | --- |
| LCGA | 1 | 580.745 | 6 | -1149.490 | -1120.098 |  |  |  |
| LCGA | 2 | 789.410 | 10 | -1558.819 | -1509.832 | 0.841 | 402.734 | <0.001 |
| LCGA | 3 | 873.692 | 14 | -1719.384 | -1650.802 | 0.648 | 162.670 | 0.102 |
| LCGA | 4 | 1018.612 | 18 | -2001.225 | -1913.048 | 0.737 | 279.704 | 0.461 |
| GMM | 3 | 1038.639 | 26 | -2025.278 | -1897.911 | 0.845 | 179.908 | 0.2398 |

Labels: LL = Log Likelihood, npar = Number of Free Parameters, AIC = Akaike’s Information Criterion, BIC = Bayesian Information Criterion, L2 = Likelihood Ratio Chi-Square, A-LRT = Lo-Mendell-Rubin Adjusted Likelihood Ratio Test., LCGA = latent class growth analysis, GMM = growth mixture model.

**Table S5. Latent Class Growth analysis class membership probability for EQ-VAS. IL: Low-Intermediate PROMs trajectory; HH: High-High PROMs trajectory; LH: Low-High PROMs trajectory.**

| **Class** | **HH** | **LI** | **LH** |
| --- | --- | --- | --- |
| **HH** | 0.810 | 0.016 | 0.174 |
| **LI** | 0.010 | 0.847 | 0.143 |
| **LH** | 0.204 | 0.080 | 0.716 |

**Table S6. Latent Class Growth analysis class membership probability for EQ-5D-3L. II: Intermediate-Intermediate PROMs trajectory; HH: High-High PROMs trajectory; LH: Low-High PROMs trajectory.**

| **Class** | **HH** | **LH** | **II** |
| --- | --- | --- | --- |
| **HH** | 0.951 | 0.027 | 0.022 |
| **LH** | 0.029 | 0.939 | 0.032 |
| **II** | 0.035 | 0.055 | 0.910 |

**Table S7. Latent Class Growth analysis class membership probability for HOOS-PS. LI: Low-Intermediate PROMs trajectory; HH: High-High PROMs trajectory; LH: Low-High PROMs trajectory.**

| **Class** | **LI** | **LH** | **HH** |
| --- | --- | --- | --- |
| **LI** | 0.864 | 0.135 | 0.001 |
| **LH** | 0.058 | 0.792 | 0.150 |
| **HH** | 0.017 | 0.175 | 0.808 |

**Table S8. Patients cross-classification according to the trajectory group for HOOS-PS and EQ-5D-3L.**

| **​​** | | **EQ-5D-3L** | | |
| --- | --- | --- | --- | --- |
|  |  | *HH* | *LH* | *II* |
| **HOOS-PS** | *HH* | 373 | 242 | 3 |
|  | *LH* | 109 | 191 | 22 |
|  | *LI* | 20 | 15 | 15 |

**Table S9. Comparison between the 15 patients cross-classified in the worst LCGA classes and the rest of the sample.**

|  | **Other patients (*n* = 976)** | **Worst performing (*n* = 15)** | **p-value** |
| --- | --- | --- | --- |
| **Sex** |  |  | 0.72 |
| Female | 501 (51%) | 7 (47%) |  |
| Male | 475 (49%) | 8 (53%) |  |
| **Age** | 60.27±13.86 | 67.33±12.93 | 0.047 |
| **BMI** |  |  | 0.756 |
| normal weight/underweight | 336 (36%) | 4 (27%) |  |
| Overweight | 395 (42%) | 7 (47%) |  |
| Obese | 209 (22%) | 4 (27%) |  |
| Missing | 36 | 0 |  |
| **ASA** |  |  | 0.012 |
| 1 | 234 (26%) | 1 (6.7%) |  |
| 2 | 519 (57%) | 7 (47%) |  |
| >=3 | 153 (17%) | 7 (47%) |  |
| Missing | 70 | 0 |  |
| **Diagnosis** |  |  | 0.254 |
| Primary coxarthrosis | 683 (70%) | 13 (87%) |  |
| Other | 293 (30%) | 2 (13%) |  |
| **In-hospital complications** |  |  | 0.074 |
| no | 972 (100%) | 14 (93%) |  |
| yes | 4 (0.4%) | 1 (6.7%) |  |
| **Incision** |  |  | 0.834 |
| Anterior | 289 (30%) | 5 (33%) |  |
| Lateral | 613 (63%) | 10 (67%) |  |
| Posterior-lateral | 66 (6.8%) | 0 (0%) |  |
| Missing | 8 | 0 |  |
| **Head diameter** |  |  | 0.759 |
| <=32 mm | 429 (44%) | 6 (40%) |  |
| >32 mm | 547 (56%) | 9 (60%) |  |
| Length of stay, days | 6.78±2.54 | 7.93±3.13 | 0.061 |

**Table S10a.** Patients who have completed the follow-up questionnaires 6 months before lockdown or during lockdown (March to May 2020).

|  | | **before lockdown** | | | | **during lockdown** | | | |  |
| --- | --- | --- | --- | --- | --- | --- | --- | --- | --- | --- |
|  | | **n** | **%** | **Mean** | **SD** | **n** | **%** | **Mean** | **SD** | **p-value** |
| **Patients** | |  |  |  |  |  |  |  |  |  |
|  | **completers** | 498 | 74.6 |  |  | 217 | 67.2 |  |  | 0.019 |
|  | **lost** | 170 | 25.4 |  |  | 106 | 32.8 |  |  |  |
| **PROMs baseline score** | |  |  |  |  |  |  |  |  |  |
| **EQ-5D-3L** | | 498 |  | 0.83 | 0.16 | 217 |  | 0.85 | 0.16 | 0.156 |
| **EQ-VAS** | | 498 |  | 77.6 | 15.5 | 217 |  | 78.7 | 15.1 | 0.297 |
| **HOOS-PS** | | 498 |  | 82.45 | 16.2 | 217 |  | 85.1 | 14.4 | 0.044 |

**Table S10b.** Patients who have completed the follow-up questionnaires 12 months before lockdown or during lockdown (March to May 2020).

|  | | **before lockdown** | | | | **during lockdown** | | | |  |
| --- | --- | --- | --- | --- | --- | --- | --- | --- | --- | --- |
|  | | **n** | **%** | **Mean** | **SD** | **n** | **%** | **Mean** | **SD** | **p-value** |
| **Patients** | |  |  |  |  |  |  |  |  |  |
|  | **completers** | 83 | 63.4 |  |  | 529 | 61.5 |  |  | 0.701 |
|  | **lost** | 48 | 36.6 |  |  | 331 | 38.5 |  |  |  |
| **PROMs baseline score** | |  |  |  |  |  |  |  |  |  |
| **EQ-5D-3L** | | 83 |  | 0.86 | 0.15 | 529 |  | 0.85 | 0.17 | 0.817 |
| **EQ-VAS** | | 83 |  | 81.8 | 12.2 | 529 |  | 78.9 | 17.5 | 0.553 |
| **HOOS-PS** | | 83 |  | 85.6 | 14.4 | 529 |  | 84.8 | 15.5 | 0.893 |
