# Supplementary material for: Patient reported outcomes measures (PROMs) trajectories after elective hip arthroplasty: a latent class and growth mixture analysis
Source: J Patient Rep Outcomes. 2022 Sep 9;6:95. doi: 10.1186/s41687-022-00503-5 (PMC9462642; doi:10.1186/s41687-022-00503-5)
Supplement: Supplementary file 2 — Additional file 2. R Script for LCGA. [file 41687_2022_503_MOESM2_ESM.docx]

Script commented

# Load the require packages

library(tidyverse) *# data analysis package*
library(haven) *# import .sav file*
library(lcmm) *# estimation of various extensions of the mixed models*

# Import dataset

We imported the dataset used for the analysis formatting the columns’ names to snake_case.

dataset <- read_sav("Data/dataset.sav") %>%
 janitor::clean_names()

# Data cleaning

The following lines of codes recode the original timestamps to 0, 1, 2 and remove the unnecessary variables.

data <- dataset %>%
 mutate(time = case_when(time == 2 ~ 0,
 time == 0 ~ 2,
 time == 1 ~ 1))

ginocchio <- data %>%
 filter(intervento_cod == 2) %>%
 select(-starts_with("hoos"))

hip <- data %>%
 filter(intervento_cod == 1) %>%
 select(-starts_with("koos"))

# Hip EQ-VAS

Latent class growth analysis for the EQ-VAS variable. We ra n models with 1 to 5 classes. Each model had 100 random starts and a 30 iterations in the optimization algorithm.

*# data formatting*
hip_eq <- hip %>%
 select(nosologico, time, eq_vas) %>% *# select the necessary variables*
 as.data.frame() *# convert the dataset to a data.frame object. Need for the hlme() function*

*# set the seed for random number generator*
set.seed(2021)

*# 1-class model to set initial start values*
hip_eq_lcga1 <- hlme(eq_vas ~ time, subject = "nosologico", ng = 1, data = hip_eq)

*# gridsearch() function used to better explore the parameter space for models with 2 to 5 classes*
*# 2 classes model*
hip_eq_lcga2 <- gridsearch(rep = 100, maxiter = 30, minit = hip_eq_lcga1, cl = 10,
 hlme(eq_vas ~ time, subject = "nosologico",
 ng = 2, data = hip_eq, mixture = ~ time))

*# 3 classes model*
hip_eq_lcga3 <- gridsearch(rep = 100, maxiter = 30, minit = hip_eq_lcga1, cl = 10,
 hlme(eq_vas ~ time, subject = "nosologico",
 ng = 3, data = hip_eq, mixture = ~ time))

*# 4 classes model*
hip_eq_lcga4 <- gridsearch(rep = 100, maxiter = 30, minit = hip_eq_lcga1, cl = 10,
 hlme(eq_vas ~ time, subject = "nosologico",
 ng = 4, data = hip_eq, mixture = ~ time))

*# 5 classes model*
hip_eq_lcga5 <- gridsearch(rep = 100, maxiter = 30, minit = hip_eq_lcga1, cl = 10,
 hlme(eq_vas ~ time, subject = "nosologico",
 ng = 5, data = hip_eq, mixture = ~ time))

Model results and trajectories visualization

*# table with results for the 5 models*
summarytable(hip_eq_lcga1, hip_eq_lcga2, hip_eq_lcga3, hip_eq_lcga4, hip_eq_lcga5,
 which = c("G", "loglik", "conv", "npm", "AIC", "BIC", "SABIC", "entropy", "%class"))

*# selecting and saving the best model*
write_rds(hip_eq_lcga3, "classi/hip_eq_lcga3.rds")

*# model summary*
summary(hip_eq_lcga3)

*# extraction of the classes probabilities and assigned class for each observation*
hip_eq_gruppi <- hip_eq_lcga3$pprob %>%
 mutate(class = as_factor(class)) %>% *# class variable recoded as categorical*
 as_tibble() *# not necessary*

*# save the table as .csv file for sharing*
write.csv(hip_eq_gruppi, "hip_eq_gruppi.csv")

*# trajectories visualization*
hip_eq %>%
 left_join(hip_eq_gruppi) %>%
 as_tibble() %>%
 group_by(class, time) %>%
 summarise(eq_vas = mean(eq_vas, na.rm = TRUE)) %>%
 ungroup() %>%
 ggplot(aes(time, eq_vas, color = class)) +
 geom_line()

The same procedure was applied for the other PROMs scales.
